# Exploring the Nutritional Ecology of Stunting: New Approaches to an Old Problem

**DOI:** 10.3390/nu12020371

**Published:** 2020-01-31

**Authors:** Daniel J. Raiten, Andrew A. Bremer

**Affiliations:** Pediatric Growth and Nutrition Branch, *Eunice Kennedy Shriver* National Institute of Child Health and Human Development, National Institutes of Health, Bethesda, MD 20892, USA; andrew.bremer@nih.gov

**Keywords:** stunting, nutrition, children, malnutrition

## Abstract

Despite a declining prevalence, stunting remains an elusive target for the global health community. The perception is that stunting represents chronic undernutrition (i.e., due to inadequate nutrient intake associated with food insecurity, low-quality diet, and suboptimal infant feeding practices in the first two years of life). However, other causes include maternal–fetal interactions leading to intrauterine growth retardation, poor maternal nutrition during pregnancy and lactation, and maternal and pediatric infections. Moreover, physical, economic, demographic, and social environments are major contributors to both food insecurity and conditions that limit linear growth. Overall, factors representing both the internal and external “nutritional ecologies” need to be considered in efforts to reduce stunting rates. Nutritional assessment requires better understanding of the mechanism and role of nutrition in growth, clear expectations about the sensitivity and specificity of the tools used, and inclusion of bio-indicators reflecting the extent and nature of the functional effect of poor nutrition and environmental factors contributing to human physical growth. We provide a perspective on current knowledge about: (i) the biology and contribution of nutrition to stunting/poor growth; (ii) our current nutritional assessment toolkit; (iii) the implications of current assessment approaches for clinical care and public interventions; and (iv) future directions for addressing these challenges in a changing global health environment.

## 1. Introduction

The global nutrition and health agenda is framed by a number of key consensus statements designed to support the community’s efforts to address specific aspects of this agenda. Each of these statements includes “targets” designed to address specific aspects of the current state of malnutrition (under- and over-nutrition). These efforts are exemplified by the World Health Organization (WHO) Global Nutrition Targets for 2025 [1] and the “United Nations Sustainable Development Goals” [2]. Prominent in both are targets to address the global burden of “stunting.” The presumption is that stunting is primarily the result of undernutrition. Is it? Do we understand the etiology and biology of stunting? If it is nutrition, which aspect of nutrition? Poor intake? Specific deficiencies of micronutrients? What is the role of the health context, e.g., concomitant infection, inflammation? How do we know?

In light of these and other questions, how valid is stunting as a trigger for public health interventions? Can the provision of food suffice to ameliorate this enduring challenge? The ability to achieve these goals will be dependent on a clear understanding of what stunting is, what its causes are, how it is assessed, and what are the best approaches for prevention and potential treatment. This concept paper will outline some of the key aspects of this critical global health challenge.

## 2. Background

Nutrition is intimately and inextricably involved in all aspects of human growth and development. The manifestations of malnutrition, primarily undernutrition, have been described for years. Among the outcomes of undernutrition most prominently described has been the impact on body composition and growth. Box 1 provides definitions for the three main types of undernutrition most often seen in children.

Box 1 **UNICEF Categories of Undernutrition in Children**

**Underweight:** Moderate—below minus two standard deviations from median weight for age of reference population; and severe—below minus three standard deviations from median weight for age of reference population.

**Wasting:** Moderate and severe—below minus two standard deviations from median weight for height of reference population.

**Stunting:** Moderate and severe—below minus two standard deviations from median height for age of reference population.

https://www.unicef.org/infobycountry/stats_popup2.html

As first delineated by Waterlow [3], undernutrition has been broadly classified as either “wasting,” which describes low weight for height/length, versus stunting, i.e., pertaining to low height/length for age [4]. This terminology has been adopted by the global health community as an indicator to both describe and serve as a trigger for programs to address undernutrition. While these terms clearly describe a perturbation in the systems involved in growth and development, in and of themselves, they are neither prescriptive nor do they provide clarity with regard to causality. In particular, stunting—which is assumed to reflect a chronic condition affecting growth and related developmental outcomes—remains a poorly understood challenge to human development. Moreover, for numerous reasons, the reliance on stunting as an indicator of poor nutrition has also come into question. Herein, we will explore some of these issues and provide some suggestions for alternative approaches.

## 3. What’s the Problem?

**Figure 1:**
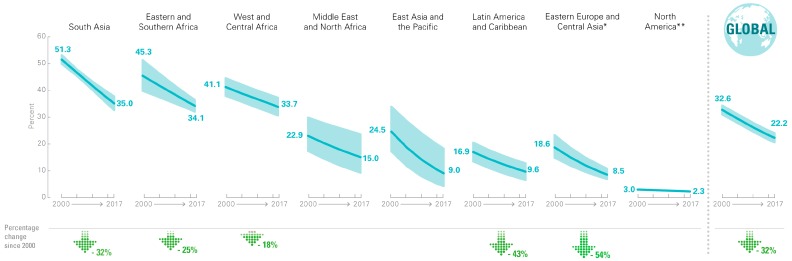
Percentage of children under 5 who are stunted, by region, 2000 to 2017 [6].

Recent trends have shown a decrease in the prevalence of stunting. In 2010, it was estimated that 171 million children (167 million in developing countries) were stunted [5]. Globally, the prevalence of childhood stunting decreased from 39.7% (95% CI 38.1, 41.4) in 1990 to 26.7% (95% CI 24.8, 28.7) in 2010 [5]. In 2017, 22.2%, or just under one in four, children aged under 5 years worldwide had stunted growth. Despite this improvement, in 2017, nearly one-third of all children living in South Asia and sub-Saharan Africa were stunted (Figure 1).

## 4. A Complex Global Health Context: Stunting, is it Only Undernutrition?

As noted, stunting is generally assumed to be a result of undernutrition. As a result, programs that rely on stunting as a trigger for intervention have primarily focused on this presumed association. However, there is substantial evidence linking stunting to numerous other adverse health conditions.

The global health context has become increasingly complex. In order to provide increased precision and to avoid unintended consequences, this complexity will demand a different approach to both assessment and interventions for conditions with potential multiple etiologies.

In 2017, the United Nations (UN) reported that the numbers of hungry people had increased for the first time in a decade, primarily as a result of conflict and climate change [7]. In addition, the prevalence of micronutrient malnutrition has not been well measured, although it is assumed that this is also a significant problem. Along with the continuing burden of infectious disease (e.g., human immunodeficiency virus [HIV]/acquired immunodeficiency syndrome [AIDS], tuberculosis [TB], malaria, diarrheal disease, and emerging infectious diseases caused by Zika, Ebola, etc.) and an exploding burden of non-communicable diseases (NCDs; e.g., obesity, type 2 diabetes, cardiovascular disease [CVD], and cancer), the relevance and impact of global health continues to become more complex. The implications of this complexity are particularly compelling because they dramatically impact the health and development of children.

Of particular relevance to this discussion is a recent analysis of the relationship between malnutrition and TB in India that documented a direct association between the two conditions [8]. However, although stunting has been linked with TB, it is generally the case that poor nutrition precedes or coincides with the infection rather than there being a demonstrable causal link. Similarly, a historical association has existed between HIV exposure, treatment, and childhood growth [9]. Recent studies have confirmed this relationship and suggest that, rather than a direct link to malnutrition, growth deficits in HIV may be metabolic consequences of the interaction between in utero exposure, maternal health, and inflammation [10,11].

In a similar manner, two concomitant conditions, diarrheal disease and environmental enteric dysfunction (EED), a complex syndrome associated with enteric inflammation and structural damage to the gastrointestinal tract [12], have been strongly associated with stunting [13,14,15]. However, the nature of the relationships is not clear at this time, other than to note that both diarrheal disease and EED are associated with inflammation. With specific regard to EED, a recent analysis suggested a role for inflammation as a plausible pathway linking the condition to stunting [16]. Interestingly, a recent report from the Biomarkers Reflecting Inflammation and Nutritional Determinants of Anemia (BRINDA) group highlighted the association between a marker of chronic (α-2 glyco-protein: AGP) but not acute (C-reactive protein: CRP) inflammation and poor linear growth in children [17].

## 5. Conundrum: What Are We Talking About?

A vernacular exists to describe various aspects of the relationships between food, nutrition, and health, and to support the development, implementation, and evaluation of interventions and programs to address these relationships. A discussion of the utility and expectations associated with these terms is relevant to our discussion of the value of stunting to the global health enterprise.

Hunger has been defined as “an uncomfortable or painful physical sensation caused by insufficient consumption of dietary energy. It becomes chronic when the person does not consume a sufficient amount of calories (dietary energy) on a regular basis to lead a normal, active and healthy life. Today, it is estimated that over 820 million people are going hungry” [18].

In addition to the United Nations Children’s Fund (UNICEF) definition of childhood undernutrition, the WHO provides an expansive definition of “malnutrition” that includes “deficiencies, excesses, or imbalances in a person’s intake of energy and/or nutrients”. With specific regard to undernutrition, the WHO refers to “4 broad sub-forms of undernutrition: wasting, stunting, underweight, and deficiencies in vitamins and minerals. Undernutrition makes children in particular much more vulnerable to disease and death” [19].

Both the definitions of hunger and malnutrition above are based on exposure. Hunger is clearly the result of an absence of food, whereas malnutrition (undernutrition) is a manifestation of hunger, but the absence of food/nutrients is not its only cause. As a biological variable, nutritional status (i.e., the reflection of the adequacy of nutrients to perform their functional roles within biological systems), affects and is affected by numerous biological processes and the health context of both individuals and populations. Stunting is used as a public health indicator of malnutrition (i.e., insufficient intake of food/nutrients), but it lacks the sensitivity or specificity needed to provide a clear explanation for causality.

The response to the narrow view of stunting as a form of malnutrition, and by inference a subset of hunger, is to provide food. The question is whether the provision of food is sufficient to either prevent or ameliorate stunting and its consequences, particularly in a world where obesity continues to rise. A recent analysis by Panjwani et al. [20] did, in fact, show a small positive impact of nutritional interventions on stunting. Similarly, other interventions, such as those focused on water and sanitation, have had a small impact [21].

Although progress has clearly been made regarding the reduction in global stunting prevalence, the core questions remain: can we address this condition with a “one-size-fits all” approach? If stunting cannot be prevented with a singular approach, then should it more appropriately be viewed as a multifactorial condition that has to be addressed as the sum of its parts? What is the value of continuing to use stunting as a public health indicator of malnutrition (quality and quantity of nutrients)? Perhaps the complexity of the stunting scenario supports the notion that stunting is a “syndrome” resulting from a myriad of risk factors [11] and, as a consequence, demands a more comprehensive ecological approach to causes, assessment, and interventions. The following sections focus on how we might move forward to advance our understanding of this complex relationship.

## 6. Public Health Nutrition: The Challenge of a “One-Size-Fits-All” Approach

As discussed, nutrition is clearly related to stunting, both in mothers as well as their children; however, to what extent remains to be determined. The assumption that stunting is only a consequence of food/nutritional insecurity leads to interventions that only target that relationship. The use of stunting as a public health trigger for improving nutrition means that even though some may benefit, others may not, and others may experience adverse outcomes due to exacerbation of other risks such as obesity or by missing the underlying cause, prolonging the problem. Experience tells us that such assumptions can be insufficient to address the problem and potentially lead to unintended consequences. The case of our efforts to address anemia and nutritional iron deficiency is informative in this regard.

The ongoing conundrum involving iron nutrition and anemia is illustrative of the challenges of a “one-size-fits-all” approach to public health nutrition. A low hemoglobin concentration has been used as a public health indicator of impaired iron status [22], even though anemia, similar to stunting, is a multifactorial problem. The traditional intervention to treat and prevent anemia has been to provide iron through various means. What we have learned is that: (i) nutritional iron deficiency, previously assumed to account for 50% of anemia [23], may only account for perhaps 25-35% of anemia in women of reproductive age and school-aged children [24]; (ii) giving iron supplements to people who do not need them may be harmful [25]; and (iii) focusing on only iron in the context of a low hemoglobin concentration misses the other causes and perpetuates anemia that has significant short- and long-term implications [26]. Nevertheless, nutritional iron deficiency remains a significant public health problem. Its assessment and our ability to identify biomarkers that can distinguish between nutritional need and physiological response remain obstacles [25]. This latter issue in particular is relevant to the question regarding the role of nutrition versus other potential causes of stunting.

## 7. The External and Internal Nutritional Ecology of Stunting

Stunting is a complex interplay between the internal and external nutritional ecologies. (“Nutritional Ecology” is the set of relationships existing between nutritional status, a biological variable representing a complex system composed of those processes involved in the ingestion, digestion, absorption, metabolism, and functional utilization of nutrients, and its surroundings or environment [27].) Components of the external nutrition ecology include dietary exposure, food systems, living environments (altitude above sea level, contamination, stressful and dangerous habitats), physical/economic/social/behavioral conditions and public health context, some of which have been recently explored [28]. The influence of the external environment (e.g., demographics, rural vs urban settings, cultural practices including infant feeding/breastfeeding, and sanitation) has long been known to be associated with stunting, and has been recently reinforced as a major determinant of stunting in high-prevalence settings [27,29]. An emerging factor is the potential role of the physical environment and climate change not only on food systems and health but also on specific aspects of nutrition [30]. More specifically, the relationship between stunting per se and these variables has come into question [31,32].

**Figure 2:**
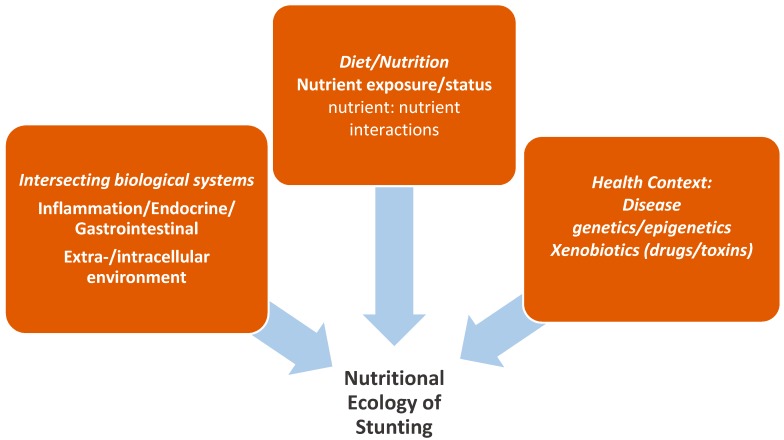
The “Internal” nutrition ecology of stunting.

In addition to external nutrition ecology, our understanding of the biology of stunting demands an appreciation of the internal nutritional ecology. Figure 2 highlights internal factors that need to be considered in the study of stunting (both biology and assessment) in order to inform our understanding of etiology and treatment.

Several fundamental questions need to be addressed regarding the application of both internal and external nutrition ecologies to advance our understanding of the role of nutrition in stunting (Box 2). In the absence of answers to these questions, our ability to intervene is severely compromised both in individuals and in populations. The complexity of the stunting scenarios demands an ecological approach that includes an appreciation of systems biology to understand the biology, translation of that biology to sensitive and specific assessment methodologies and interventions, and ultimately, to improve clinical and public health outcomes.

Box 2 **Key Questions Re: The Biology of Stunting**

1. What is the biology of stunting?
  (a) What is the role of intersecting biological systems, e.g., neuroendocrine, GI/metabolic, inflammatory, etc.?
  (b) What is the relationship between wasting and stunting?
    - Why do some children who are wasted/“malnourished” become stunted while others do not?
    - Wasting is associated with poor linear growth, but the effects of remediation on growth are mixed [33].
2. Why do some children who are born small-for-gestational age (SGA) become stunted while others do not?
  (a) 20% of stunting and 30% of wasting was reported to have prenatal origins [34].
3. Is there a trigger that causes children to go down this path, and if so, what is it?
  (a) Specific nutrient (single or multiple) deficits?
  (b) Inflammation?
  (c) Body habitus and related metabolic factors: Fat mass and related metabolomics mediated by compounds such as leptin?
  (d) Is stunting a reflection of a chronic condition? Is there a role for infection? NCDs?
  (e) Obesity: role of maternal body mass? Children who are stunted are susceptible to obesity but children who are obese do not appear to be susceptible to stunting. Is there something to be learned from our evolving understanding of the developmental origins of health and disease (DOHaD) paradigm?
4. What is the role of genetics, either in terms of prediction or adaptation to environmental conditions?
5. Assessment: What is the value of anthropometry beyond an observation of something gone wrong? How can we better translate what we know about the biology of stunting and its causes to better assessment approaches?
  (a) It has been suggested that mid-upper arm circumference (MUAC) may be an indicator of the metabolic sequelae associated with relevant changes in body compositions and the relationship to stunting [35]. Is this a viable hypothesis?
  (b) What biomarkers should we employ to increase our precision both in terms of diagnosis and interventions clinically, in the field and at scale?

## 8. Differences Between Wasting and Stunting: Beyond a Definition of “Malnutrition”

As noted above, in an attempt to provide some clarity regarding the definition of malnutrition and its implications, a number of classification schemes and definitions have been promulgated. According to Golden [36], “two types of responses have been identified when a child’s intake of an essential nutrient is insufficient—either there is continued growth whilst the body uses up the nutrient resulting in specific deficiency signs, or there is reduced growth while the tissue concentration of the nutrient is maintained.” Golden [37,38] further refined this by classifying nutrients based on their functional ramifications: Type I nutrients, which have specific responses (e.g., scurvy responsive to vitamin C, beriberi responsive to thiamine, xeropthalmia responsive to vitamin A); and Type II nutrients, which result in more generalized system failures (e.g., reduced growth due to a lack of protein and/or zinc).

Similarly, Waterlow [39] described a commonly used categorization of malnutrition:

- Type A: “a deficit in weight-for-height, represents a fairly acute state of malnutrition where growth, as shown by height, has been reasonably satisfactory until some acute episode, infective or nutritional, supervenes.”
- Type B: “a deficit in height-for-age, undernutrition over a long period has caused a retardation in linear growth.”

A simplistic view of these observed outcomes is that they are differentiated by either duration of malnutrition (Type A being more “acute”) and/or a metabolic trigger (Type B) resulting in linear growth retardation. This classification is clearly not satisfactory in terms of causal pathway or specificity. Both contributed to confusion about the distinction between “weight loss” and poor linear growth, which of course can occur simultaneously (i.e., both describe a condition associated with reduced weight for age but neither distinguishes between the physiology associated with loss of body mass versus reduced linear growth and its concomitants). Thus, Waterlow suggested use of the terms “wasting” and “stunting” to provide further clarity to the distinction between weight loss and poor growth.

For numerous reasons, the use of anthropometry to define and distinguish between wasting and stunting (i.e., reduced weight for age z-score [WAZ] versus height for age z-score [HAZ]) as public health indicators of “malnutrition,” offers several advantages. As public health indicators, both provide clear signals of a problem in the external system. They are also relatively convenient, non-invasive measures for assessing health. These terms have been codified and continue to be used to represent malnutrition and as guides for program development [40]. However, implicit in such schemes is: (i) the view of malnutrition as a result of nutrient insufficiency/exposure; and (ii) a lack of accountability for other causes of malnutrition (e.g., specific nutritional metabolic consequences of disease), and (iii) a single-nutrient perspective that does not reflect the role and nature of potential nutrient interactions with each other or within biological systems. Using this type of classification scheme can only address the “external” nutrition ecology from a macro-level and limits the ability to explore the “internal nutrition ecology,” (i.e., diet and health/disease relationships at a micro- or biological systems level). This conundrum may explain why we can show a reduction in the prevalence of stunting while some countries still maintain prevalence above the currently accepted reference values.

There are several core issues specifically relating to the challenges about how to define and respond to the intractable problem of global stunting. The presumption in many circles is that stunting is synonymous with dietary deficiency of macro- and/or specific micro-nutrients, and thus represents a continuum of malnutrition that goes from wasting to stunting, a notion recently reinforced by a large cohort analysis in the Gambia [41]. Relevant to this perspective, some of the specific challenges to address stunting include:

- Despite recent progress, there is a persistent stunting prevalence of 20%–25% among children globally;
- Stark regional differences in the prevalence of stunting continue to exist, which may reflect the local external ecology perhaps as much or more than the biology;
- The utility of stunting as a public health “trigger” for initiation of interventions may be limited due to the lack of fundamental understanding of its biology;
- In a world of exploding nutrition-related NCDs, the lack of specificity of nutritional interventions and the role of early life exposure to the development of these chronic diseases raises concerns about the clinical or public health utility of current interventions to address stunting (i.e., is it simply a matter of too much or too little food?);
- Limitations of current biomarkers to distinguish between specific nutrition-related problems and other potential causes or correlates of stunting.

Ultimately, wasting and stunting share common risk factors (i.e., elements in both the internal and external nutrition ecologies). However, at some point the manifestations of these risk factors clearly diverge [42]. As such, with respect to stunting, it is important to identify the best ways to determine what is responsible for that divergence, how to assess it, and how to use that knowledge to develop more targeted interventions to prevent it.

## 9. Principles in Nutritional Assessment: How Can We Determine the Role of Nutrition in Stunting?

Our ability to more precisely define a role for nutrition in the etiology, prevention, or treatment of stunting will require accurate and reliable approaches to nutritional assessment. This section will apply a set of principles that should be utilized in the assessment of nutrition and its role in stunting.

Nutrients can provide substrate (i.e., the material for cellular function and tissue growth), be involved in regulatory systems, and be involved in multiple systems related to growth (i.e., bone development, immunology, fat metabolism, and endocrine systems); moreover, multiple systems rely on multiple nutrients. The goals of assessment are to determine the best types and amounts of evidence that fully integrate and address the roles of diet and nutrition in all aspects of health promotion, disease prevention, and treatment in order to:

- Support the safe and effective application of existing standards of clinical care, or to establish new standards;
- Provide the requisite data to support the development and evaluation of programs, policies, and guidance;
- Ensure the validity and reliability of research data and their appropriate translation.

**Figure 3:**
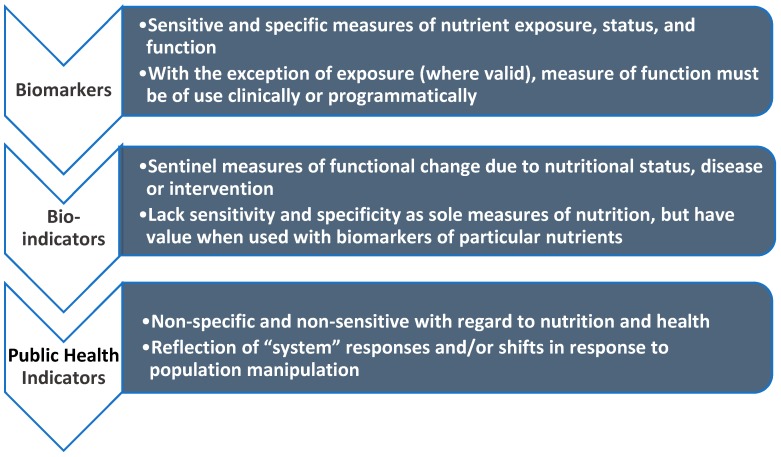
Tools for defining malnutrition (from Raiten and Combs) [43].

Nutritional assessment includes a number of tools that exist for defining malnutrition including (Figure 3):

- Public health indicators;
- Bio-indicators;
- Biomarkers.

Although each of these categories is used to define malnutrition, they all represent different realities and expectations in their ability to achieve that objective. Public health indicators reflect perturbations in the external ecology reflecting larger systems (e.g., economic, social/demographic, etc.), whereas biomarkers are sensitive and specific reflections of the internal nutritional ecology. Bio-indicators exist at the interface of these two and can reflect either the external or internal ecology and relevant systems. Growth/anthropometry is a bio-indicator that reflects perturbations in either the external (i.e., socio-economic, food/nutritional insecurity) or internal (i.e., nutritional status and/or systems involved in linear growth) ecology, or both. But clearly growth/anthropometry is not a biomarker of nutrition, and in the absence of sensitive and specific biomarkers of nutritional status, it does not provide any useful insights into the biology of nutrition that may underlie this putative outcome.

As the problem of poor growth clearly involves multiple systems and multiple nutritional inputs, it demands an approach that includes an appreciation of this complexity. Multiple etiologies affecting multiple systems have been suggested, including:

(1) Genetics: underlying adaptation to adverse environments;
(2) Environmental adjustment to low oxygen pressure due to altitude above sea level and contaminated environments;
(3) Neuroendocrine control of bone health/linear growth;
(4) Intrauterine growth retardation;
(5) Poor infant feeding practices, particularly breastfeeding;
(6) Maternal (e.g., HIV) and/or child infections;
(7) Gastrointestinal disorders [14] affecting:
  - Gastrointestinal function;
  - Microbiome composition and function;
  - Nutrient absorption;
(8) Interactions between lean body mass and fat mass;
(9) Inflammation and the immune system.

The intersection of nutrition and the inflammatory response has received considerable recent attention. Raiten et al. [44] provided a comprehensive overview of (1) the nature of the reciprocal relationships between nutrition and the immune response (each affects and is affected by the other), and (2) the importance of accounting for the presence of inflammation in the choice and interpretation of biomarkers of nutrient status. More specifically, Bourke et al. [45] reviewed the role of immune dysfunction as both a cause and an outcome of malnutrition.

The application of new technologies is a key component of both identifying causal pathways and the application of more sensitive and specific assessment methodologies that integrate that knowledge. A relevant example was recently presented by Mahmud et al. [46] in their review of the value of characterizing the metabolome/lipidome of malnourished children for the assessment of the biology of stunting.

Although a better understanding of the biology of stunting is needed, what is clear is that focusing on the outcome (i.e., poor growth) rather than the etiologies may not be the best public health strategy and certainly lacks the precision needed to develop targeted nutrition-specific or sensitive interventions. Our ability to understand causal pathways will be complicated by the fact that focusing on one aspect of this ecology misses other key components and may lead to inadequate solutions to this complex problem.

## 10. Conclusions

Significant progress has been made toward reducing the global prevalence of stunting and this will undoubtedly continue as public health efforts to address risk factors associated with this condition continue. However, our ability to truly have an impact on the prevention and treatment of stunting will demand a different level of understanding to generate the data needed to improve our approaches to this complicated condition and its risks, and to develop safe and effective programs, guidance, and standards of care.

The validity of stunting as both a public health indicator and a bio-indicator of malnutrition has recently come under increased scrutiny [31,47,48]. Using stunting as a classification scheme for defining malnutrition and thereby motivating public health interventions highlights the wider importance of poor nutrition but will not provide the precision needed to either understand, prevent, or treat stunting.

Fundamentally, there is a distinction between hunger, food insecurity, and malnutrition. Hunger is a reflection of the external nutrition ecology resulting in limited access and availability of high-quality foods to meet dietary needs. Malnutrition is about biology and the intersections of health and disease with factors relating to the internal nutritional ecology. The merging of these outcomes interferes with the ability to fully appreciate and resolve the antecedents of either. Box 3 contains a list of critical research gaps that will need to be addressed to get us where we need to go.

Box 3 **Stunting—A Complicated Research Agenda**

- Stunting: what is it (nutritional, physiological, genetic, environmental)? How do we know and how can that knowledge be collected to inform public policy?
- If nutritional, what nutrients (protein, specific amino acids, calories, zinc)?
  ○ What is the pattern of nutrients involved in those systems controlling linear growth (i.e., the “nutriome” of growth) and how can we best understand their normal interactions and what happens during fluctuations (e.g., poor status of either single or multiple nutrients)?
- How do we identify/measure stunting (anthropometry versus sensitive and specific biomarkers) clinically and in field settings, and interpret those results in the context of an increasingly complex global health context?
- Is stunting amenable to remediation?
  ○ Remediation of growth deficits, is catch-up growth possible?
  ○ If catch-up growth is possible, is it physiologically useful?
  ○ How do we define stunting?
  ○ What is the primary cause? What is the nature of the relationships between stunting and its associated conditions (e.g., neurodevelopment, development of NCDs, etc.)? Are the relationships causative or correlative?
  ○ Neurodevelopmental outcomes: these are less clear. Neurodevelopment presents a daunting array of complications, not least of which is that, depending on when and for how long the insult(s) occur, there are critical periods beyond which remediation is not possible.
- Long-term health: obesity and other NCDs:
  ○ What are the biological connections?
  ○ Is stunting a reliable predictor?

We can take solace in the recent successes resulting in the reduction in the global prevalence of stunting. Clearly efforts to improve the well-being of the human society, including reduction of hunger and malnutrition, have had a positive impact. However, the absence of answers to the questions posed above limits our ability to intervene both in individuals and in populations. The complexity of stunting scenarios demands an ecological approach that includes an appreciation of systems biology to understand the biology and to facilitate the translation of that biology to sensitive and specific assessment methodologies and interventions, with the ultimate goal of improving clinical and public health outcomes.
